# Magnesium phosphate cements in regenerative dentistry: from biomaterial design to clinical translation

**DOI:** 10.3389/fdmed.2026.1888093

**Published:** 2026-07-16

**Authors:** Nishmitha N. Hegde, Harshitha Somanatha, Chaithra Lakshmi, Niranjan Harikrishna, Mithra N. Hegde

**Affiliations:** 1 Nitte (Deemed to be University), AB Shetty Memorial Institute of Dental Sciences (ABSMIDS), Department of Conservative Dentistry and Endodontics, Mangalore, India; 2 Research Lab, AB Shetty Memorial Institute of Dental Sciences, Nitte (Deemed to be University), Mangaluru, Karnataka, India

**Keywords:** biomaterials, bone regeneration, endodontics, magnesium phosphate cement, osteogenesis, regenerative dentistry

## Abstract

Magnesium phosphate cements (MPCs) are emerging biomaterials in regenerative dentistry, offering rapid setting, high early mechanical strength, biodegradability, osteogenic potential, and intrinsic antimicrobial activity. Compared with conventional calcium phosphate cements (CPCs), MPCs demonstrate superior biological interactions and faster scaffold resorption, positioning them as promising candidates for endodontic, periodontal, craniofacial, and bone tissue engineering applications. This Mini Review critically appraises the chemistry, biological properties, clinical applications, and translational challenges of MPCs, highlighting current evidence gaps and emerging research directions. While preclinical evidence is encouraging, exothermic setting reactions, ammonia release in ammonium-containing formulations, and the absence of robust multicentre clinical trial data remain the principal barriers to widespread adoption. Advances in formulation engineering, additive manufacturing, and composite scaffold design represent the most promising avenues for addressing these limitations and facilitating clinical translation.

## 1 Introduction

Regenerative dentistry has undergone considerable evolution, driven by biomaterials that support tissue repair, osteogenesis, angiogenesis, and functional integration with host tissues (1). Calcium phosphate cements (CPCs) and calcium silicate-based materials, such as mineral trioxide aggregate (MTA), have been the primary biomaterials investigated for pulp capping, root-end filling, guided bone regeneration, and periodontal defect repair (2). Despite their established clinical track record, these materials are limited by prolonged setting times, insufficient early mechanical strength, slow degradation kinetics, and limited intrinsic antimicrobial activity characteristics that constrain performance in applications demanding rapid scaffold-tissue integration and infection control (3).

MPCs are formed through acid-base reactions between magnesium oxide (MgO) and phosphate salts, producing rapidly hardening materials with high early mechanical strength, biodegradability, and enhanced biological activity (4, 5). Magnesium is the fourth most abundant cation in the human body, playing essential roles in mineral metabolism, enzyme activation, and skeletal development. Magnesium ions (Mg^2^⁺) stimulate osteoblastic differentiation through RUNX2 activation, TRPM7-mediated transport, integrin alpha5beta1 signalling, IGFBP5 activation, and Wnt/*β*-catenin pathway stimulation (5–7), collectively underpinning the osteogenic potential of MPC-based biomaterials. Crucially, MPCs additionally demonstrate intrinsic antimicrobial activity, a property absent from most conventional dental cements, attributable to alkaline pH, osmotic stress, and Mg^2^⁺-mediated disruption of bacterial membranes (8, 9).

**Figure 1:**
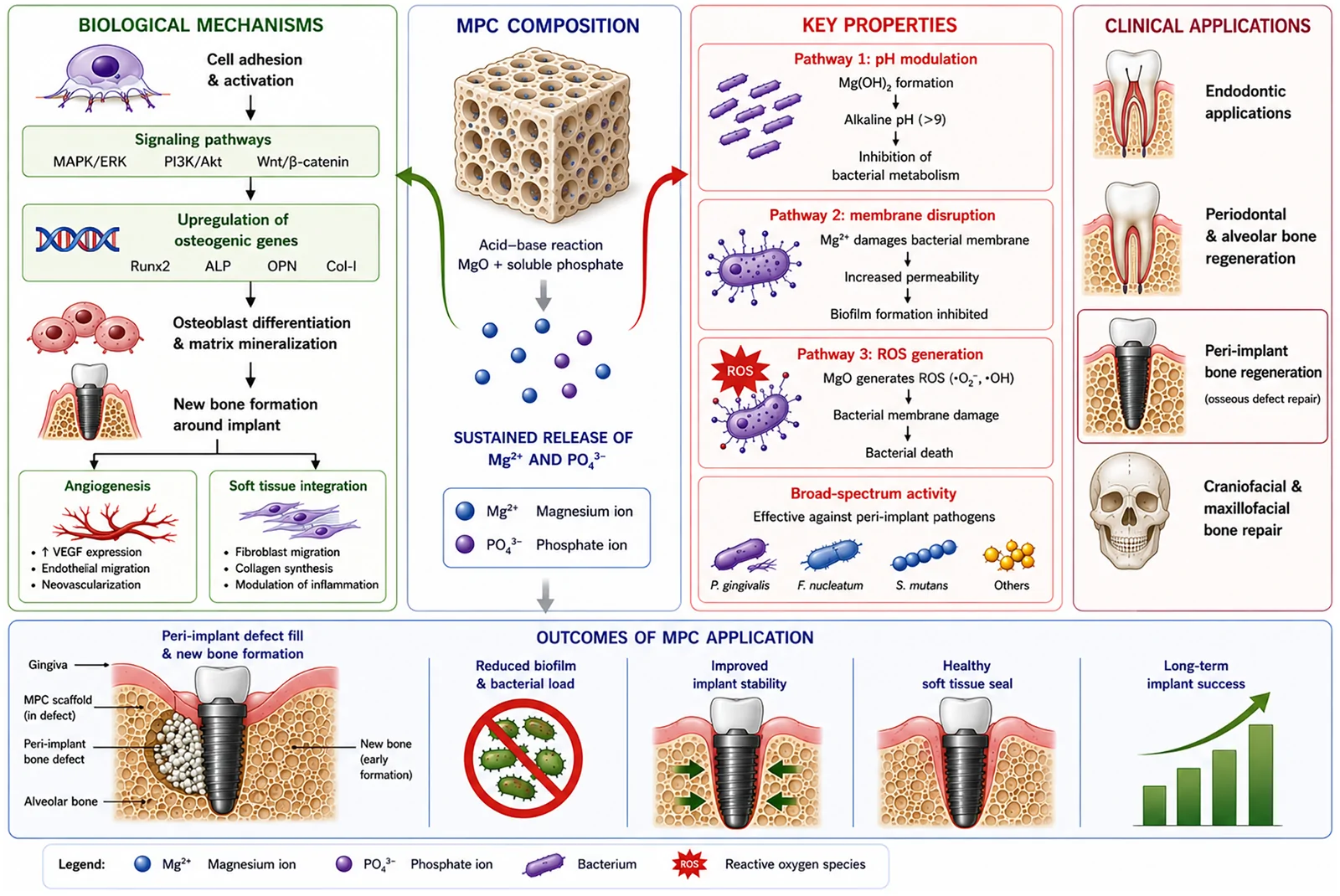
Schematic overview of MPCs in regenerative dentistry. Legend: Schematic overview of magnesium phosphate cements (MPCs) in regenerative dentistry, illustrating composition, key properties, clinical applications, current limitations, and future research directions for clinical translation.

This Mini Review critically appraises MPC research in regenerative dentistry, addressing chemical and mechanical properties, biological interactions, clinical applications, current limitations, and future research priorities, with emphasis on strategies to advance clinical translation (Figure 1).

## 2 Chemical and mechanical properties

### 2.1 Composition and setting chemistry

MPCs are typically synthesised through acid-base reactions between MgO and a soluble phosphate salt, ammonium dihydrogen phosphate (NH₄H₂PO₄) in ammonium-based systems or potassium dihydrogen phosphate (KH₂PO₄) in potassium-based systems. The reaction produces crystalline struvite (NH₄MgPO₄·6H₂O) or potassium struvite (KMgPO₄·6H₂O) as the principal cementing matrix. Clinically acceptable setting times of 5–10 min are substantially shorter than most CPCs. Hydration kinetics and final cement properties are governed by MgO particle size and purity, the Mg/P molar ratio, curing temperature, and the phosphate precursor (9, 10).

### 2.2 Mechanical strength

A principal advantage of MPCs is high early mechanical strength. Mestres and Ginebra (2011) reported compressive strengths of approximately 30 MPa at one hour and 50 MPa at 24 h across multiple MPC formulations, substantially exceeding apatitic CPCs, which achieve only ∼1 MPa at one hour and ∼35 MPa at maximum (9). Optimised potassium phosphate-based crystalline formulations have achieved values exceeding 100 MPa (11, 12), generally surpassing cancellous bone and most conventional CPCs. Both amorphous and crystalline matrices achieve comparable compressive strengths at 24 h, suggesting that the degree of matrix consolidation may be more important than crystallinity for early mechanical performance (9).

### 2.3 Exothermic reactions and mitigation

The setting reaction of MPCs is exothermic. Sustained temperatures above 42–47 °C risk irreversible damage to the dental pulp and surrounding tissues. Several mitigation strategies have been identified: glucose coating of MgO particles to slow dissolution kinetics (13); borax and boric acid retardation through B₄O₇^2^⁻ adsorption onto MgO surfaces (14, 15); substitution of ammonium phosphate with KH₂PO₄ or NaH₂PO₄, which eliminates ammonia generation and moderates the exothermic profile (9); trimagnesium phosphate filler incorporation to dilute reactive MgO (16); and calcium silicate compounding, which buffers reaction kinetics and improves bioactivity (17).

### 2.4 Degradation behaviour

MPCs are biodegradable, with resorption kinetics governed by porosity, crystallinity, Mg/P ratio, and biopolymeric additive content. Higher porosity and lower crystallinity accelerate dissolution, while chitosan incorporation moderates fluid ingress and slows degradation (18, 19). Synchronisation of MPC degradation rate with the rate of new bone deposition is a central formulation design challenge: excessively rapid resorption compromises structural continuity before mineralised matrix bridges the defect, while slow degradation impedes vascular ingrowth and cell migration (19, 20). Mg^2^⁺ released during degradation stimulates alkaline phosphatase activity, upregulates RUNX2 and osteocalcin, and suppresses osteoclastogenesis through RANKL pathway inhibition, shifting the resorption-formation balance in favour of new bone deposition (6, 7) (Table 1).

**Table 1:** Comparative properties, biological performance, clinical applications, and formulation challenges of MPCs versus CPCs in regenerative dentistry.

Property	MPC	CPC	Clinical Relevance	Reference
Setting time	5–10 min	15–60 min	Critical for surgical workflow	(5, 9)
Compressive strength (24 h)	∼50 MPa (conventional); >100 MPa (optimised potassium systems)	∼1–35 MPa	Load-bearing applications	(9, 11, 12)
Antimicrobial activity	Intrinsic; superior to MTA and Ca(OH)₂ in planktonic models	Limited intrinsic activity	Endodontic infection control	(8, 25)
Osteogenic potential	High; Mg^2^⁺-mediated RUNX2, Wnt/*β*-catenin, TRPM7 activation	Moderate; osteoconductive scaffold only	Bone regeneration	(6, 7, 22)
Biodegradation	Intermediate; tunable	Slow; poorly resorbable	Scaffold replacement by bone	(19, 20)
Exothermic setting	Significant (ammonium); mitigated in potassium/retarder-modified systems	Minimal	Pulp vitality safety	(9)

MPC, magnesium phosphate cement; CPC, calcium phosphate cement; MTA, mineral trioxide aggregate.

## 3 Biological properties and composite formulations

### 3.1 Osteogenic potential and cellular response

The osteogenic potential of MPCs is mediated primarily through sustained Mg^2^⁺ release, activating multiple intracellular signalling cascades. Studies employing dental pulp stem cells (DPSCs), periodontal ligament stem cells (PDLSCs), and mesenchymal stem cells (MSCs) have demonstrated Mg^2^⁺-induced upregulation of RUNX2 and alkaline phosphatase at concentrations of 1–10 mM (6, 7). IGFBP5 activation and integrin-mediated mechanotransduction pathways have also been implicated (7, 21). Although methodological heterogeneity across published *in vitro* studies with respect to cell type, Mg^2^⁺ concentration, extraction medium, and incubation duration constrains direct cross-study comparison, preliminary evidence consistently supports the osteogenic capacity of MPC-derived ionic environments.

*In vivo* studies in rat calvarial defect and rabbit femoral condyle models have demonstrated favourable biocompatibility, limited inflammatory response, and progressive scaffold resorption coupled with new bone deposition (22, 23). MPCs enhance vascularisation through Mg^2^⁺-mediated upregulation of HIF-1*α* and VEGF in endothelial progenitor cells, alkaline microenvironment-driven angiogenic signalling, and macroporous scaffold architectures that provide physical channels for capillary ingrowth (20, 24).

### 3.2 Antimicrobial properties

MPCs exhibit intrinsic antimicrobial activity without the need for exogenous antibiotic incorporation. The mechanism involves four pathways: high osmolarity causing bacterial plasmolysis (25); alkaline pH denaturing membrane proteins (9); Mg^2^⁺ competing with essential divalent cations at membrane binding sites; and ionic imbalance impairing transmembrane transport and ATP synthesis (8). Comparative planktonic studies have demonstrated larger inhibition zones and improved antibacterial efficacy relative to MTA and calcium hydroxide (8, 25). Gram-negative organisms may be more susceptible due to their thinner peptidoglycan layer. Critically, planktonic models do not replicate the protective extracellular polymeric substance (EPS) matrix of structured oral biofilms, within which bacteria exhibit markedly increased tolerance to ionic and osmotic antimicrobial mechanisms. Long-term antibiofilm behaviour of MPCs, therefore, remains inadequately characterised and constitutes a research priority.

### 3.3 Bioactive additives and composite formulations

Incorporation of bioactive additives tailors the biological, mechanical, and handling characteristics of MPCs. Bioactive glass fibres improve fracture toughness through crack-bridging while releasing silicate, calcium, and phosphate ions to stimulate osteogenic differentiation (26). Calcium silicate compounding enhances compressive strength, modulates degradation, and improves bioactivity through sustained silicate ion release (17). Chondroitin sulfate improves matrix cross-linking and promotes angiogenesis through VEGF-mediated signalling (27, 28). Zinc incorporation stimulates osteogenic differentiation through BMP-2 signalling and confers additional antimicrobial activity (29). Magnesium-containing microspheres promote M2 macrophage polarisation, creating a pro-regenerative immunological microenvironment (30). Handling additives, including collagen peptides and chitosan modify paste viscosity, injectability, and degradation kinetics without substantially altering mechanical performance (4, 31). Collectively, these composite modification strategies confirm MPCs as a highly tunable biomaterial platform.

## 4 Clinical applications

### 4.1 Endodontic applications

MPCs have been investigated for pulp capping, pulpotomy, apexification, perforation repair, and root canal obturation (3). MPCs demonstrate dentin bonding strengths superior to MTA, attributable to controlled setting expansion that promotes micromechanical interlocking within dentinal tubules, reducing interfacial microleakage (8, 32). Caution is warranted with ammonium-based formulations in direct pulp capping, given the risk of thermal injury at temperatures exceeding 42–47 °C; retarder-modified and potassium phosphate-based systems with attenuated exothermic profiles are preferable candidates for vital pulp therapy (9, 16). Long-term sealing ability studies demonstrate maintained marginal integrity over extended observation periods (32). Additive manufacturing has enabled 3D-printed MPC-based endodontic scaffolds with patient-specific root canal geometries, representing a significant advance in anatomical precision for regenerative endodontic procedures (33).

### 4.2 Periodontal and alveolar bone regeneration

The combination of osteogenic potential, antimicrobial activity, and controlled biodegradability makes MPCs well-suited to periodontal bone defect regeneration. Injectable MPC formulations incorporating foaming agents, calcium carbonate, and citric acid generate macroporous architectures *in situ* following delivery, promoting cell infiltration and vascularisation (34). In animal models of periodontal bone defect, MPC-based cements have demonstrated faster bone fill and superior mineralisation density compared to conventional CPCs (22, 23). In comparative studies across craniofacial and orthopaedic bone repair applications, MPCs consistently demonstrate superior early mechanical performance, faster bone regeneration, and advantageous resorption-regeneration kinetics relative to CPCs (4, 11, 23).

## 5 Discussion

### 5.1 Current limitations and controversies

Several challenges continue to limit the clinical translation of MPCs. Exothermic setting reactions, particularly in ammonium-based formulations, threaten pulp vitality, while ammonia release remains a cytotoxicity concern in ammonium phosphate systems. Premature scaffold degradation can compromise mechanical stability if resorption outpaces osteogenesis. The translational relevance of current preclinical findings is constrained by animal models that do not fully replicate the oral environment, including salivary dynamics, polymicrobial biofilms, and masticatory loading. Substantial methodological heterogeneity in Mg/P ratios, phosphate precursors, retarder systems, scaffold architectures, animal models, and outcome measures further impedes direct comparison of findings and may contribute to overestimation of treatment effects due to predominance of positive preclinical reports and publication bias. Most importantly, robust clinical evidence remains scarce, with only limited human data and short follow-up periods available.

### 5.2 Formulation engineering and scaffold advances

Addressing material-level limitations has motivated a coherent body of formulation engineering work. The conceptual framework is the decoupling of advantageous biological and mechanical properties from undesirable thermal, chemical, and degradation characteristics through targeted modification of setting chemistry and matrix architecture. Potassium and sodium phosphate formulations eliminate ammonia generation while achieving mechanical properties broadly comparable to ammonium-based systems (9). Retarder-modified and trimagnesium phosphate-filled formulations have demonstrated substantially attenuated exothermic profiles within clinically acceptable ranges (14, 16).

Additive manufacturing, specifically extrusion-based 3D printing of MPC pastes, uniquely combines patient-specific geometric customisation from medical imaging data with reproducibly controlled pore architecture for guided vascularisation and bone ingrowth (20, 33).Growth factor loading of 3D-printed calcium phosphate cement scaffolds has been demonstrated as an effective strategy to augment osteogenic and angiogenic potential (35), and gelatin incorporation into additively manufactured MPC scaffolds has been explored to improve structural integrity and handling characteristics (36) Integration of resorbable polymer-ceramic composite systems, including PCL-ceramic frameworks, represents an approach applicable to MPC compounding that extends the mechanical performance envelope of biodegradable scaffold platforms (37).

### 5.3 Future research priorities

Several research priorities must be addressed to facilitate clinical translation. The most immediate need is adequately powered multicentre randomised clinical trials evaluating MPC-based interventions across endodontic, periodontal, and craniofacial indications. Parallel comprehensive long-term safety assessments, including biocompatibility testing in accordance with ISO 10993 standards, are required, with particular attention to thermal effects, ammonia-associated cytotoxicity, and the consequences of prolonged Mg^2^⁺ exposure. Standardisation of formulation protocols and outcome reporting frameworks is equally important to enable meaningful cross-study comparisons and evidence synthesis. Future research should prioritise the development of multifunctional MPC composites with validated antibiofilm activity against complex polymicrobial oral biofilms, as planktonic efficacy data cannot be directly extrapolated to clinically relevant biofilm environments (29, 38). Development of stimuli-responsive MPC-based biomaterials capable of programmable Mg^2^⁺ release in response to pH changes, enzymatic activity, or mechanical loading may enable more precise regulation of the local healing environment. Integration of MPCs with stem cell therapies and immunomodulatory strategies, particularly those promoting M2 macrophage polarisation, represents a promising avenue for enhancing tissue regeneration and directing host responses toward favourable healing outcomes (2, 30).

## 6 Conclusion

MPCs represent a compelling addition to the biomaterial toolkit for regenerative dentistry. Their combination of rapid setting, high early mechanical strength, intrinsic antimicrobial activity, and Mg^2^⁺-mediated osteogenic and angiogenic signalling addresses several key limitations of existing CPC and bioceramic systems. The principal limitations of exothermic setting, ammonia release, and premature scaffold resorption are technically tractable, and mitigation strategies have been demonstrated at the laboratory and small-animal preclinical scale. These preclinical demonstrations provide a sufficient basis for progression to controlled clinical investigation. The absence of robust multicentre human clinical trial data remains the most consequential barrier to translation and defines the central research agenda for the field in the coming decade.

## Data Availability

All supporting data will be provided on reasonable request.
